# Persistent STAT5 Phosphorylation and Epigenetic Dysregulation of GM-CSF and PGS2/COX2 Expression in Type 1 Diabetic Human Monocytes

**DOI:** 10.1371/journal.pone.0076919

**Published:** 2013-10-18

**Authors:** Erin Garrigan, Nicole S. Belkin, John J. Alexander, Zhao Han, Federica Seydel, Jamal Carter, Mark Atkinson, Clive Wasserfall, Michael J. Clare-Salzler, Matthew A. Amick, Sally A. Litherland

**Affiliations:** 1 Department of Pathology, Immunology, and Laboratory Medicine, College of Medicine, University of Florida, Gainesville, Florida, United States of America; 2 Sanford-Burnham Medical Research Institute Orlando, Orlando, Florida, United States of America; La Jolla Institute for Allergy and Immunology, United States of America

## Abstract

STAT5 proteins are adaptor proteins for histone acetylation enzymes. Histone acetylation at promoter and enhancer chromosomal regions opens the chromatin and allows access of transcription enzymes to specific genes in rapid response cell signals, such as in inflammation. Histone acetylation-mediated gene regulation is involved in expression of 2 key inflammatory response genes: *CSF2,* encoding granulocyte-macrophage colony stimulating factor (GM-CSF), and *PTGS2,* encoding prostaglandin synthase 2/cyclooxygenase 2 (PGS2/COX2). Prolonged *CSF2* expression, high GM-CSF production, and GM-CSF activation of *PTGS2* gene expression all are seen in type 1 diabetes (T1D) monocytes. Persistent phosphorylation activation of monocyte STAT5 (STAT5Ptyr) is also found in individuals with or at-risk for T1D. To examine whether elevated T1D monocyte STAT5Ptyr may be associated with aberrant inflammatory gene expression in T1D, blood monocytes from non-autoimmune controls and T1D patients were analyzed by flow cytometry for STAT5Ptyr activation, and by chromatin immuno-precipitation (ChIP) analyses for STAT5Ptyr’s ability to bind at *CSF2* and *PTGS2* regulatory sites in association with histone acetylation. In unstimulated monocytes, STAT5Ptyr was elevated in 59.65% of T1D, but only 2.44% of control subjects (p<0.0001). Increased STAT5Ptyr correlated with T1D disease duration (p = 0.0030, r^2^ = 0.0784). Unstimulated (p = 0.140) and GM-CSF-stimulated (p = 0.0485) T1D monocytes, had greater STAT5Ptyr binding to epigenetic regulatory sites upstream of *CSF2* than control monocytes. Increased STAT5Ptyr binding in T1D monocytes was concurrent with binding at these sites of STAT6Ptyr (p = 0.0283), CBP/P300 histone acetylase, acetylated histones H3, SMRT/NCoR histone deacetylase (p = 0.0040), and RNA Polymerase II (p = 0.0040). Our study indicates that in T1D monocytes, STAT5Ptyr activation is significantly higher and that STAT5Ptyr is found bound to *CSF2* promoter and *PTGS2* enhancer regions coincident with histone acetylation and RNA polymerase II. These findings suggest that the persistent activation of STAT5 by GM-CSF may be involved in altering the epigenetic regulation of these inflammatory response genes in T1D monocytes.

## Introduction

Myeloid antigen presenting cells (APC) are the primary immune regulatory cells inducing and enforcing peripheral self-tolerance. Granulocyte Macrophage Colony Stimulating Factor (GM-CSF) is a major differentiation and inflammatory response cytokine that is both produced by and acts on myeloid APC, including peripheral blood monocytes, interstitial macrophages, and myeloid dendritic cells [1]–[3]. APC dysfunction is a key component of immunopathology of multiple autoimmune diseases, including Type 1 diabetes (T1D) [4], [5].

Chronic high prostaglandin synthase 2 (PGS2/COX2) and GM-CSF expression by T1D patient monocytes and nonobese diabetic (NOD) mouse myeloid cells allow for the production of high levels of the pro-inflammatory prostanoid prostaglandin E2, PGE2. PGE2 blocks myeloid APC ability to prevent auto-reactive T cell escape from activation induced cell death [6]–[9]. Excessive PGE2 also supports the chronic inflammatory environment. Such a chronic inflammatory environment promotes immune cell tissue infiltration; and in T1D, eventual pancreatic beta cell destruction [8]–[10]. In autoimmune myeloid cells, GM-CSF induces persistent activation of the signal transduction/epigenetic enzyme adaptor proteins, STAT5A and STAT5B. As a consequence, dimerized phosphorylated STAT5A/B (STAT5Ptyr) binds to regulatory sites upstream of the *CSF2* gene and to enhancer sequences found between and possibly within the *IL10* to *PTGS2* gene region on Chromosome 1 (mouse and human) [7], [10]–[15]. In this study, we examined the effects of elevated human monocyte STAT5Ptyr in T1D on its binding at regulatory regions associated with *CSF2* and *PTGS2.*

High PGS2/COX2 expression seen in at-risk human subject monocytes is directly correlated to T1D risk and is indicative of a 1.5-fold increase in disease susceptibility within the high risk population [6], [16]. This biomarker for T1D risk is detectable very early in life, but is difficult to quantify above the inherent background of prostanoid activation due to blood collection and processing, (e.g., activation of *PTGS2* mRNA in monocytes by adherence to glass or endotoxin contamination within 90 min *ex vivo*). High GM-CSF production is readily detectable in primary culture of T1D monocytes, but is less specific for T1D and autoimmune dysfunction than PGS2/COX2 [7], [11], [15]. In contrast, activation of STAT5A and STAT5B by GM-CSF is both selective for autoimmune myeloid cell dysfunction and readily analyzable using modification specific antibodies directed against tyrosine phosphorylated isoforms of these proteins (anti-STAT5Ptyr (Tyr694/699)) [11], [15], [17], [18].

In this report, we use a multiplex format chromatin immunoprecipitation (ChIP) assay that allows us to simultaneously detect several components of the cells epigenetic and transcriptional regulatory protein machinery associated on the same specific regulatory sequences upstream of *CSF2* and *PTGS2* genes. Using these tools, we investigate the mechanism of how STAT5Ptyr could affect epigenetic regulation of both GM-CSF and PGS2/COX2 expression. Our findings indicate that STAT5Ptyr in T1D but not non-autoimmune monocytes respond to GM-CSF stimulation by binding to the promoter of *CSF2* and in an enhancer region associated with the Chromosome 1 region between *IL10* and *PTGS2.* ChIP evaluation of GM-CSF-stimulated STAT5Ptyr binding at these sites show coincidence with histone modification and recruitment of epigenetic and transcription proteins that can modulate epigenetic gene expression in chromatin regions.

## Materials and Methods

### Human Blood Sample Collection

All work done for this study involving the participation of human volunteers was done in accordance with IRB approved protocols (University of Florida and Sanford-Burnham Medical Research Institute IRB approved protocol UF372-1996) and only with informed consent. After giving written informed consent, healthy volunteers and T1D patients donated small blood samples either by venipuncture (up to10 ml) or finger prick (<0.5 ml), during routine visits to the pediatric and adult endocrinology clinics at the Shands at UF hospital on the University of Florida, Gainesville, FL campus. Information of the subject populations is given in **Table 1**. Overall demographics of the sample population were reflective of the client population served by Shands at UF Hospital. Control group inclusion criteria required participants to be free of diabetes, autoimmune diseases, and without a family history of autoimmunity or diabetes. T1D patients were enrolled from practices of clinical faculty associated with Shands at UF Hospital and were considered in overtly hyperglycemic or with a known history of abnormal glucose tolerance testing at the time of sample collection. Blood samples were collected into heparinized tubes, coded and de-identified of all HIPAA defined personal medical information prior to laboratory processing. Blood samples were either processed immediately for analysis at the University of Florida or shipped on wet ice to arrive within 24 hr of the draw at the Sanford-Burnham Medical Research Institute facilities in Orlando, Florida, for ChIP and flow cytometric analyses.

**Table 1:** Human Subject Population Characteristics for Peripheral Blood Samples collected over the 3 year study period (2006–2009).

Sample group	# of Individuals Sampled§(age range in years)	# Males(age range in years)	# Females(age range in years)	Mean Duration of Disease (in years)
**Healthy Controls** *	37	20	17	−
	(newborn- 47 yrs; median23.0, mean 24.9, SD 8.2 yrs)	(10–45 yrs; median 23.0, mean24.6, SD 6.1 yrs)	(newborn-47 yrs; median 23.0,mean 25.1, SD 10.3 yrs)	
**T1D Patients** †	53	25	28	Range: New onset to 38 years
	(6–69 yrs; median 16.0, mean18.6, SD 11.0 yrs)	(10–51 yrs; median 14.0.0, mean16.0, SD 9.4 yrs)	(6–69 yrs; median 19.0, mean22.1, SD 12.3 yrs)	Median 7.6 yrs Mean 8.9 yrs; SD 7.5 yrs

* individuals with no overt symptoms of disease and with no family history of T1D or other autoimmune diseases.

† Individuals with overt hyperglycemia or abnormal GTT/FPIR

§ Most individuals were analyzed only one time and the assay run in duplicate for each sample

### Flow Cytometric Analysis for STAT5Ptyr in Peripheral Blood Monocytes

The flow cytometric analyses were completed using a small volume, whole blood modification of our previously described intracellular STAT5Ptyr analysis using modification specific antibodies for tyrosine 694/699 phosphorylated STAT5 [11]. Blood samples were diluted 1∶1 with FACS Buffer (1X PBS without Calcium or Magnesium (Cellgro, MediaTech, VWR), pH 7.2, 1% RIA grade BSA (Sigma-Aldrich Chemical, St Louis, MO), 0.1% Na Azide (Fisher Scientific, cell culture/reagent grade, Pittsburgh, PA); 0.22micron filter sterilized) and held at room temperature (rt, approx. 25°C). Anti-CD14 antibody conjugates (FITC, PE, or PerCP, BD Biosciences) and non-specific mouse IgG antibodies for Fc blocking were added and the samples incubated in the dark for at least 15 min at rt. After incubation, each sample was diluted 1∶1 with Fix N′ Perm (BD Biosciences) or 4% formaldehyde in 1XPBS pH 7.5 and then held at rt for at least 10 min. The samples were then equally divided between 2 FACS polystyrene tubes (Falcon 4570, BD Biosciences) and 1 ml of Saponin Buffer (1X PBS without Calcium or Magnesium (Cellgro, MediaTech, VWR), pH 7.2, 1% RIA grade BSA (Sigma-Aldrich), 0.1% Na Azide (Fisher Scientific), 0.5% Saponin (Sigma-Aldrich); 0.22micron filter sterilized) was added to each aliquot. The sample aliquots were centrifuged at 600× g for 5 min, rt and the supernatants decanted off to leave approximately 200 µl of fluid to re-suspend the cells. One microgram protein of either mouse non-specific IgG isotype-APC conjugate control antibodies or specific anti-STAT5Ptyr-APC conjugate antibodies were then added to the aliquots for intracellular staining (UpState/Millipore, Temecula, CA; both control and specific antibodies were conjugated with APC-Phyto-Link kit and clean up column from Prozyme, San Leandro, CA). After at least 45 min incubation in the dark at rt, the samples are washed repeatedly by centrifugation with a total of 2–4 ml of Saponin buffer, until red cell hemoglobin contamination was diminished. The samples were then re-suspended in a total of 400 µl of FACS Buffer and held at 4°C in the dark for analysis within the next 24 hr. Consistent with previous analyses, no significant difference in the % CD14+ cells was seen between controls and T1D samples [6], [16]. The percentage STAT5Ptyr+/CD14+ monocytes out of 5000–10000 events collected in each sample group were compared by ANOVA and Mann-Whitney U or Student t test comparisons using Prism 5/6 software (GraphPad Software, Inc, La Jolla, CA).

### Human Sequence Analysis

**Figure 1:**
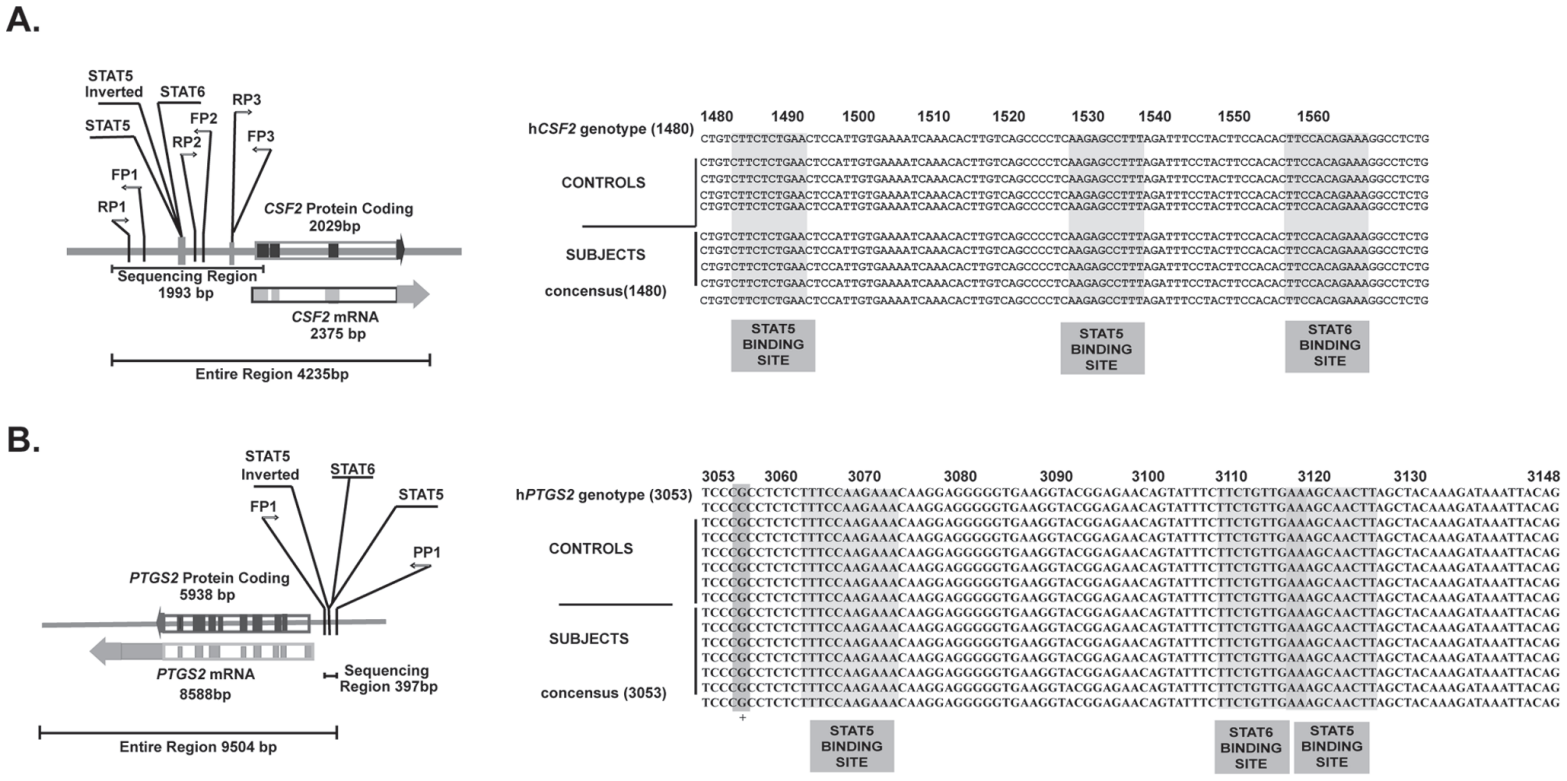
T1D Patient/Control Sequence Analysis for Upstream Regulatory Regions of the *CSF2* and *PTGS2* genes. A. Schematic representation and representative sequence data of the human promoter region upstream of *CSF2,* the gene encoding GM-CSF. The location of the amplified region is delineated by forward and reverse primers, FP1 and RP1, respectively. Also defined are the STAT5 and STAT6 binding sites (boxed) within the region. No T1D-specific polymorphisms have been detected in the 24 samples tested. **B.** Schematic representation and representative sequence data of the enhancer region upstream of the PGS2/COX2 gene, *PTGS2.* The location of the amplified region is delineated by forward and reverse primers, FP1 and RP1, respectively. Also defined are the STAT5 and STAT6 binding sites within the region (boxed). No T1D-specific polymorphisms have been detected in the 24 samples tested.

Twelve non-autoimmune controls and twelve T1D patient samples were subjected to sequence analysis. Population characteristics for this test population are given in **Table 2**. Primers specific to the *CSF2* promoter and *PTGS2* enhancer regions in the human genome that are homologous to the regions in the mouse genome were designed using Net Primer (**Figure 1**). Primers for the enhancer region upstream of the *PTGS2* gene (5′GGGGCGAGTAAGGTTAAGAAAGGC; 3′ ACATTTAGCGTCCCTGCAAATTCTG, Sigma-Genosys, Sigma-Aldrich) select for a region approximately 397 bp in length and include two STAT5 binding sites [13]. The primers designed to amplify the GM-CSF gene (*CSF2*) promoter (5′GTGGATTGGAAAGACTTGTTGACTG; 3′ TTCACATGCTCCCAGGGCT, Sigma-Genosys, Sigma-Aldrich) generate a PCR product of length 1993 bp. DNA samples were purified using Qiagen Blood and Cell Culture DNA Mini Kit and amplified by PCR using Eppendorf 2.5X Master Mix (Fisher Scientific) and either *CSF2* promoter or *PTGS2* enhancer primers. PCR amplification products were visualized on 1% agarose gel (SeaKem Fisher Scientific) and purified using DNA Clean and Concentrator TM-5 (Zymo Research, Orange, CA) then amplified again in a Big Dye PCR amplification reaction (Applied Biosystems, Foster City, CA) and sequenced using an AB capillary sequence analyzer (Applied Biosciences, Foster City, CA) and found comparable to sequences reported in these analyses are available in publically accessible NIH gene libraries.

**Table 2:** Characteristics of patient, control and relative samples collected for *CSF2* and *PTGS2* sequencing analysis.

Characteristic	Healthy Controls (n = 24)	T1D Patients*(n = 24)
**Gender**		
**Male**	15	12
**Female**	9	12
**Age Range**	4.97–44.89	6.56–35.1

* Individuals with overt hyperglycemia or abnormal GTT/FPIR; mean disease duration = 8.5 years ±7.65 SD.

### Primary Cell Culture of Blood Monocytes

Peripheral blood mononuclear cells (PBMC) were isolated out of 10 ml peripheral blood or umbilical cord blood samples from 4 non-autoimmune controls and 10 T1D patients, using a ficoll histopaque gradient (Sigma-Aldrich). The PBMC were washed with 1×PBS (Cellgro, MediaTech, VWR) and re-suspended in sterile RPMI medium (Cellgro, MediaTech, VWR), supplemented with 10% fetal calf serum (FCS, Cellgro, MediaTech, VWR), and 1% antibiotic-antimycotic mix (PSA:100X penicillin, streptomycin, amphylterin mix; Cellgro, MediaTech, VWR) to a density of 1 to 5×10^6^ cells per well and cultured for 1 hr at 37°C/5% CO_2_ to allow for monocytes adherence. The cultures were then washed with sterile 1× PBS (Cellgro, MediaTech, VWR) to remove non-adherent cells and re-fed with RPMI 10%FCS/1%PSA medium alone, or with 5.6 nM human recombinant GM-CSF (Invitrogen, Carlsbad, CA), 500 U/ml human recombinant IL-4 (Invitrogen), 1 µg/ml dexamethasone (Sigma-Aldrich) or 1 ng/ml endotoxin (LPS) (Sigma-Aldrich). Dexamethasone (DEX) and LPS stimulations were included in the analyses as potential ‘negative’ and ‘positive’ controls, respectively, for STAT activation and epigenetic regulation of transcription, based on previous reports [10], [13], [14]. These adherence-purified cultures were typically 65–85% monocytes by CD14^+^ flow cytometric analysis. Cultures were held 2 hr at 37°C/5%CO_2_ to allow time for activation by LPS or suppression by DEX, then washed with 1×PBS and then lysed *in situ* using ChIP Lysis Buffer (1% SDS, 10 mM EDTA, 50 mM Tris, pH 8.1; Sigma-Aldrich reagents) supplemented with a proteinase inhibitor cocktail (Roche, Indianapolis, IN). The lysates were sonicated on ice to shear chromatin complexes to approximately 1000 bp lengths and frozen at −80°C until used for ChIP analysis.

### Multiplex ChIP Analysis for STAT-mediated Epigenetic Function

Monocyte cell extracts were analyzed as individual (not pooled) samples. Thawed extracts were pre-cleared of non-specific DNA and Protein G binding proteins by incubation *in situ* with salmon sperm-Protein G coated sepharose beads (UpState/Millipore) and bead-free supernants aliquoted equally over 8 PCR microtubes in a strip/capped format (Eppendorf, Westbury, NY). The aliquots were diluted with ChIP Dilution Buffer (16.7 mM Tris-HCl, pH 8.1, 0.01% SDS, 1.1% Triton X-100, 1.2 mM EDTA, 167 mM NaCl; Sigma-Aldrich & Fisher Scientific reagents) to a total volume of 200 µl each. Salmon Sperm-Protein G sepharose beads (Upstate/Millipore) were pre-incubated with 1 µg/30 µl beads suspension in a 96-tube ‘Multiplex’ format using parallel ChIP isolations with one tube of each of the following UpState/Millipore ChIP certified antibodies: anti-STAT5Ptyr, anti-STAT6Ptyr, anti-RNA Polymerase II, anti-acetylated Histone H3, anti-P300/anti-CBP acetylase, and anti-SMRTe deacetylase. Thirty microliters of the antibody-bead mixes were added to 7 of the 8 tubes of sample, with the final tube being left unprecipitated to act as a total DNA control. After overnight incubation at 4°C, ChIP bead complexes were collected via centrifugation and washed successively with 200 µl of each of the following: Low Salt buffer (20 mM Tris HCl, pH 8.1, 0.1% SDS, 1%Triton X-100, 2 mM EDTA, 150 mM NaCl; Sigma-Aldrich & Fisher Scientific reagents), High Salt buffer (20 mM Tris HCl pH 8.1, 0.1% SDS, 1% Triton X-100, 2 mM EDTA,500 mM NaCl), LiCl buffer (0.25 M LiCl, 1% IGEPAL-CA630, 1% deoxycholic acid, 1 mM EDTA, 10 mM Tris HCl, pH 8.1; Sigma-Aldrich & Fisher Scientific reagents), and TE buffer (10 mM Tris HCl pH 8.0, 1 mM EDTA; Sigma-Aldrich & Fisher Scientific reagents). The beads were then resuspended in freshly prepared 0.1 M sodium bicarbonate/1% SDS buffer (Fisher Scientific) and incubated for 45 min at rt to uncouple the ChIP complexes from the beads. The beads were removed by centrifugation and sodium chloride added to resultant supernatant for a final concentration of 500 mM NaCl (Fisher Scientific). The salted samples were then incubated for 4 hr at 65°C to reverse the formaldehyde crosslinking. After incubation, 0.5 M EDTA pH 8.0 (Sigma-Aldrich), 1 M Tris HCl pH 6.5 (Fisher Scientific, and Proteinase K, Qiagen, Valencia CA) were added and the samples incubated for 1 hr at 45°C to digest the remaining proteins. The resultant chromosomal DNA samples were either analyzed as is or further purified by either phenol/chloroform extraction or with QIAGEN genomic DNA kit. Real time PCR (RLT PCR) analyses were done as previously described for single ChIP isolations using primers specific for the promoter region of the *CSF2* gene and the enhancer region of the *PTGS2* gene [10], [13], [14], [18]. The results of the RLT PCR were compared for each specific antibody used in the multiplex ChIP analyses as an R value, where R = 2^(ΔCt^_non-specific IgG_^−ΔCt^_specific Ab_^)^ [19]. When cultures of less than 4×10^6^ cells were used to make ChIP extracts, a second, re-amplification round of RLT PCR with the same primers was often needed to verify the results. Such low sample volumes and re-amplication led to less conclusive results in the *PTGS2* analysis of 2 control and 5 T1D samples, and as a result were not included in the data analysis presented.

## Results

### T1D Subject Monocytes have Significantly Higher STAT5 Activation than Healthy, Non-autoimmune Controls

**Figure 2:**
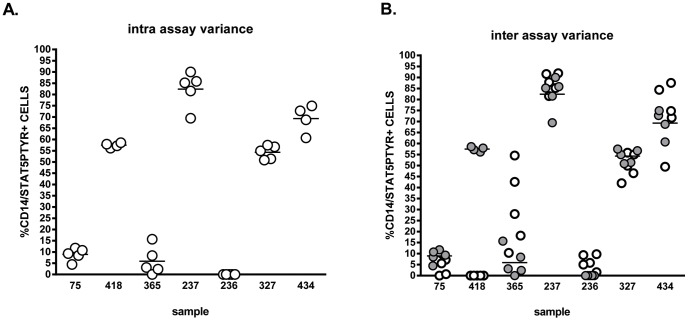
Intra- & Inter-assay Variance for the flow cytometric analysis of STAT5Ptyr in human peripheral blood monocytes. Whole blood from T1D (#418, 237,327, and 434) and non-autoimmune healthy controls (75, 365, and 236) was analyzed for STAT5 activation as described in the Methods & Materials. Each sample aliquot was analyzed 4 to 5 times during each run of the analyses. **A. Intra-assay Variance**: There was an assay variance of <5% in between control samples and intra-assay from 0 to 6.3% for a given control sample’s analysis; whereas, there was an assay variance of <13% between T1D samples with an intra-assay 1 to 7.9% for a given T1D sample’s analysis. There was a significant (>13-fold) difference between the % STAT5Ptyr/CD14+ cells detected in the T1D samples tested (mean 65.85%, SD 12.79%, n = 4) as compared to controls analyzed in the same assays (mean 4.952%, SD 4.547%, n = 3). **B.** **Inter-assay** **Variance:** Each sample analyzed 24 hr to 48 hr after shipment and storage at 4°C. An inter-assay variance of 2-6-fold between 24 hr (filled circles) and 48 hr (clear circles) assay runs of 3 control samples and a 1.1-fold variance in 3 T1D samples was seen between 24 and 48 hrs. At 24 hr, there was a significant (>13-fold, *p = 0.0017) difference between the % STAT5Ptyr/CD14+ cells of T1D samples (mean 65.85%, SD 12.79%, n = 4) as compared to controls (mean 4.952%, SD 4.547%, n = 3) in same assays. However, this significant difference decreased to 5.4-fold at 48 hr (*p = 0.013), due to the increased inter-assay variability seen in control samples after storage.

Blood samples from 37 non-autoimmune, healthy controls and 53 T1D patients (**Table 1**) were assessed for STAT5 activation in monocytes without *ex vivo* stimulation using intracellular flow cytometry. Intra-assay and inter-assay variance in this modified assay were assessed on samples drawn 24 hr prior to analysis and samples shipped on ice and then stored for over 72 hr post draw. There was an assay variance of <5% between individual control samples and intra-assay variance from 0 to 6.3% for a given control sample’s analysis. The assay variance between individual T1D samples was <13%, with an intra-assay 1 to 7.9% for a given T1D sample’s analysis (**Figure 2a**). There was a significant (>13-fold, p = 0.0006) difference between the % STAT5Ptyr/CD14+ cells detected in the T1D samples tested (mean 65.85%, SD 12.79%, n = 4) and that of non-autoimmune controls analyzed in same assays (mean 4.952%, SD 4.547%, n = 3) (**Figure 2a**). In sample aliquots analyzed after 24 hr shipping and storage at 4°C, there was inter-assay variance between 24 hr and 48 hr assay runs of 2 to 6-fold for 3 control samples and 1.1-fold in 3 T1D samples (**Figure 2b**). There was a significant (>13-fold, p = 0.0017) difference between the % STAT5Ptyr/CD14+ cells detected in the T1D samples tested (mean 65.85%, SD 12.79%, n = 4) and that of non-autoimmune controls (mean 4.952%, SD 4.547%, n = 3) analyzed in the same assays at 24 hr. However, this significant difference decreased to 5.4-fold at 48 hr (p = 0.0130), and was lost at 72 hr post draw (data not shown) due to the increased intra-assay variability seen in control samples after storage.

**Figure 3:**
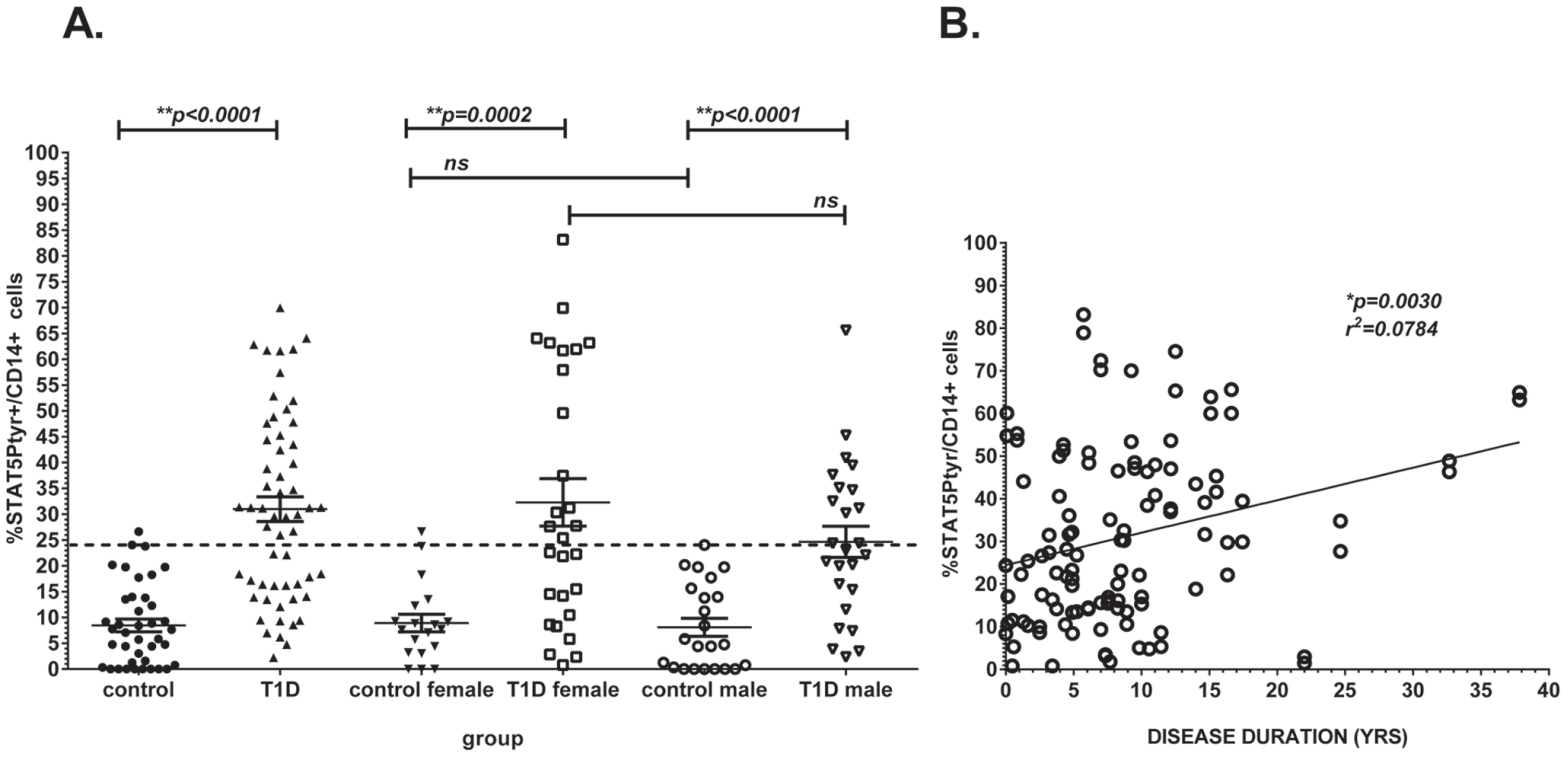
STAT5 Phosphorylation in CD14+ Peripheral Blood Monocytes and Correlation of STAT5Ptyr with Duration of Disease. **A.** Unactivated monocytes from 53 T1D patients had significantly higher activated STAT5 without *ex vivo* activation than monocytes from 37 healthy, non-autoimmune controls as analyzed by intercellular flow cytometry using small volume (<100 µl) whole peripheral blood samples (control mean 8.46%±7.83 SD, T1D 30.97%±18.00 SD, p<0.0001, Mann Whitney U test). Gender did not affect these data, though a trend for higher values in female T1D monocytes was seen: control females mean 8.904% ±7.445 SD, n = 17 vs. control males mean 8.904% ±8.242 SD, n = 20, (p = 0.6463, Student t test); female T1D subjects 32.30% ±24.37 SD; n = 28, vs.T1D male subjects 24.62% ±15.07 SD; n = 25, (p = 0.4820, Mann-Whitney U test). Overall, 59.65% of T1D subjects had %STAT5Ptyr/CD14 levels at or above the control mean +2 standard deviations (24.12%; indicated by the dashed line) compared to 2.44% of controls. **B.** No correlation with age was seen in any of the test groups; however, a trend for increasing %STAT5Ptyr levels with age was noted. This trend revealed a significant correlation of %STAT5Ptyr levels with disease duration within the T1D subject group (p = 0.0005, linear regression, R^2^ value = 0.0784). Due to the very low sample volumes tested, most but not all samples were analyzable in duplicate from a single random blood draw. Data points on graph depict average of replicates for each individual.

Using this analysis method to test a larger sample population, we found that unactivated monocytes from T1D patients had significantly higher activated STAT5 without *ex vivo* activation than monocytes from healthy, non-autoimmune controls, as detected in small volume (<100 µl) whole peripheral blood samples stored up to 24 hr at 4°C (control mean 8.460%±7.833SD, T1D 30.97%±18.00, p<0.0001, pair-wise Mann Whitney U test, **Figure 3a**). Overall, nearly 60% (59.65%) of T1D subjects, but less than 3% (2.44%) of controls, had %STAT5Ptyr levels at or above the control mean +2 standard deviations (24.15%; indicated by the dashed line, **Figure 3a**). These findings parallel our previously reported analyses in larger volume (>1 ml) fresh blood samples [11].

### Population Factors Affecting STAT5 Activation in Peripheral Blood Monocytes

Control cell samples had significantly lower %STAT5Ptyr levels compared to T1D subjects (p<0.0001, Mann Whitney U test, **Figure 3a**). Female control monocytes had the same %STAT5Ptyr levels compared to male control monocytes (mean 8.094%±7.445SD control females versus mean 8.094%±8.242 control males, p = 0.6463, Student t test). There was no significant difference (p = 0.4840, Mann-Whitney U test) but a slight trend for higher %STAT5Ptyr levels between female T1D subjects (32.30%±24.37 SD) and that of T1D male subjects (24.62% ±15.07 SD).

Though there was a trend for increased STAT5Ptyr levels in older T1D subjects, there was no statistically significant correlation found with age for %STAT5Ptyr levels in monocytes within any of the test groups (data not shown). However, there was a significant correlation of STAT5Ptyr levels increasing with disease duration in the T1D subject group (p = 0.0030, r^2^ = 0.0784, **Figure 3b**).

### STAT Binding Sites in the Upstream Regulatory Regions of CSF2 and PTGS2 genes

Unlike our previous findings in NOD mice [18], no T1D-specific polymorphisms were detected in either *CSF2* or *PTGS2* upstream regulatory regions that directly associated with the increased GM-CSF production or COX2/PGS2 expression seen in at-risk/T1D individuals [7], [11]. However, multiple STAT5 binding sites were found in these regions in close proximity to or overlapping STAT6 binding sites (**Figure 1**). If excess activated STAT5 binding could interfere with STAT6 binding at both the *CSF2* promoter and the *PTGS2* enhancer, it might enhance STAT5Ptyr response to cytokine activation by GM-CSF or IL-2, and/or hinder immunosuppression STAT6Ptyr activation by IL4 [10], [12], [20]–[23]. To test for this possibility, we developed a multiplex format ChIP assay to simultaneously analyze STAT5Ptyr, STAT6Ptyr, acetylase (CBP/P300), and deacetylase (SMRT/NCoR) binding, histone H3 acetylation, and the presence of RNA Polymerase II on or near the STAT5/STAT6 binding sites in the *CSF2* and *PTGS2* regulatory regions.

**Figure 4:**
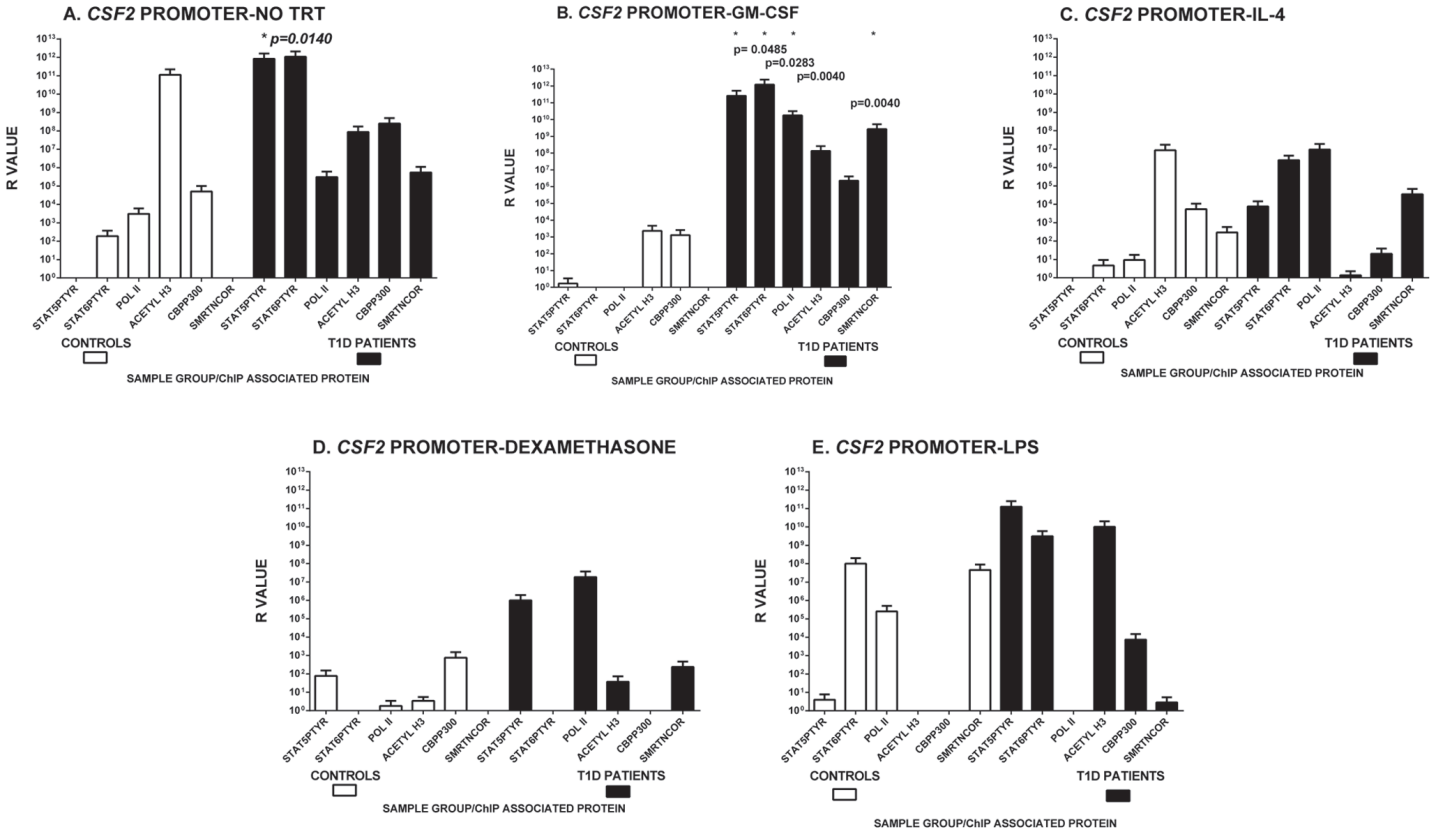
Multiplex ChIP analysis of Protein Binding, Epigenetic Modification, and Transcription at the *CSF2* promoter region. Peripheral blood monocytes were isolated and incubated at 37°C/5%CO_2_ for 2 hr in (**A**) media alone (0) or continuously supplemented with (**B**) 5.6 nM GM-CSF (GM-CSF), (**C**) 500 U/ml IL-4 (IL4), (**D**) 100 µM dexamethasone (DEX), or (**E**) 1 ng/ml endotoxin (LPS). Time of incubation was set to allow the negative (DEX) and positive (LPS) control supplements adequate time to affect the cells. The cells were washed and extracted for ChIP analysis as described in the Materials and Methods and used in multiplex ChIP Analysis of STAT5Ptyr, STAT6Ptyr, acetylase (P300), and deacetylase (SMRTe) binding, histone H3 acetylation, and the presence of RNA Polymerase II on or near the STAT5/STAT6 binding sites in the *CSF2* promoter region shown in **Figure 3A**. ChIP multiplex analyses depicted represent the mean of 3 to 5 runs of the analyses (± SEM) on each of control and T1D samples of peripheral blood monocytes. Data is presented on a log scale as an R value = 2^(ΔCt^_nonspecific IgG_^−ΔCt^_specific Ab_^)^ for each specific antibody used [19]. Individual samples (not pooled) were run in at least duplicate and represent data from 10 T1D, and 4 Control group members. Asterisks indicate where significant differences were seen between binding found in T1D monocytes and that found in non-autoimmune control monocytes (Mann-Whitney U test, p values listed on the graph and in Results text).

We used this assay to profile epigenetic modification and transcription potential in isolated monocytes from 10 T1D and 4 healthy, non-autoimmune control subjects without stimulation or after *ex vivo* stimulation with GM-CSF, and IL-4 (**Figures 4** **&** **5**). The results of these analyses suggest that without any exogenous stimulation, T1D subject monocytes exhibit significantly more STAT5Ptyr binding (p = 0.0140, Mann-Whitney U test) at the *CSF2* promoter, and a trend for epigenetic modification, and potential for transcription at both *CSF2* and *PGTS2* loci than non-autoimmune healthy controls. Unstimulated STAT6Ptyr binding in T1D monocytes was not outcompeted by STAT5Ptyr at either site, but was able to bind equally as well as STAT5Ptyr to this sequence. In comparison, little to no STAT5Ptyr and STAT6Ptyr binding were seen to either *CSF2* or *PTGS2* associated regulatory sites without stimulation in control monocytes (**Figures 4A** **&** **5A**).

**Figure 5:**
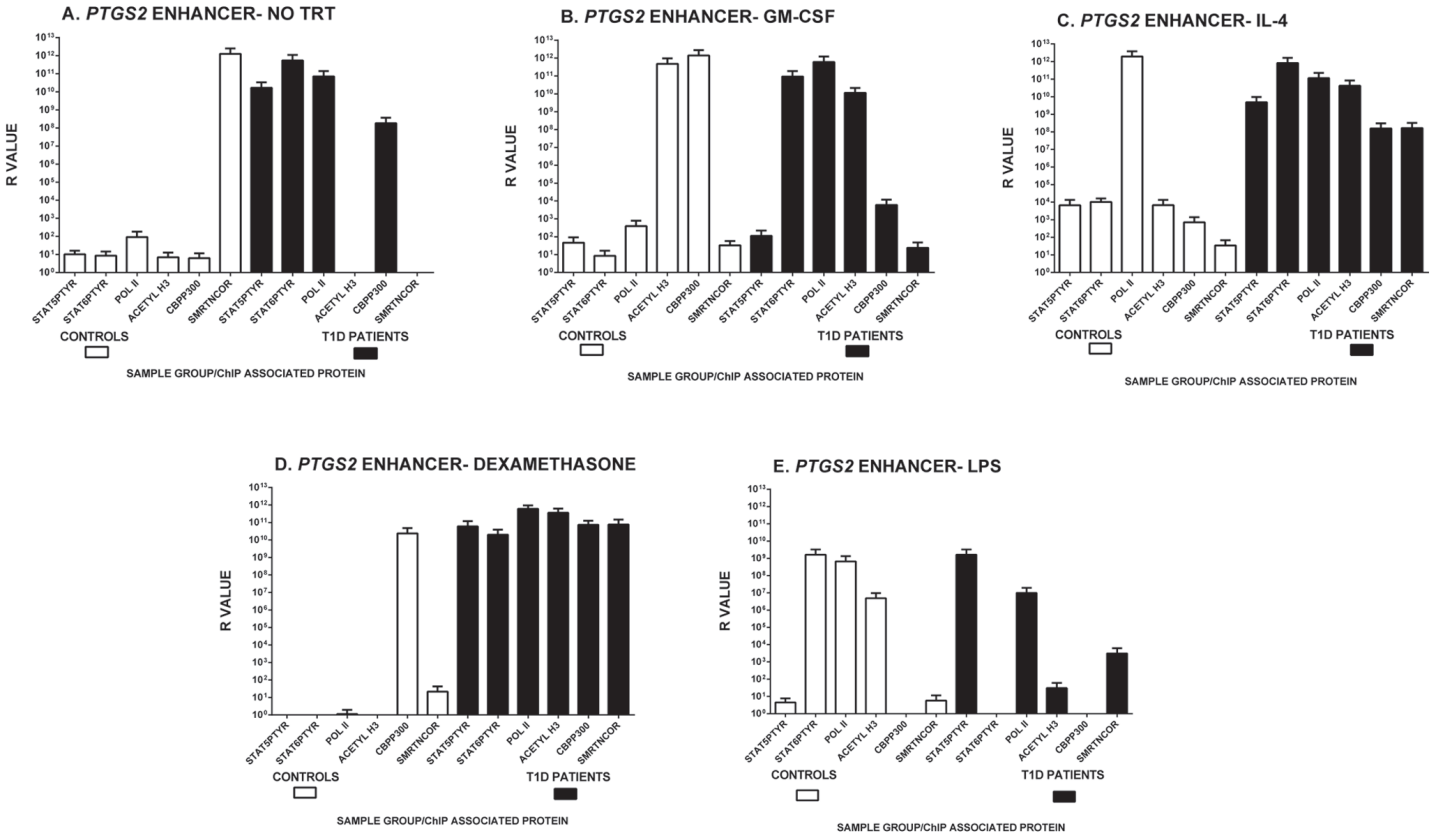
Multiplex ChIP analysis of Protein Binding, Epigenetic Modification, and Transcription at the *PTGS2* enhancer region. Peripheral blood PBMC monocytes were isolated and incubated at 37°C/5%CO_2_ for 2 hr in the continuous presence of (A) media alone (0) or media supplemented with (B) 5.6 nM GM-CSF (GM-CSF), (C) 500 U/ml IL-4 (IL4), (D) 100 µM dexamethasone (DEX), or (E) 1 ng/ml endotoxin (LPS). This timeframe is used to allow for optimization of the negative (DEX) and positive (LPS) control stimulation. The cells were washed and extracted for ChIP analysis as described in the Materials and Methods and used in multiplex ChIP Analysis of STAT5Ptyr, STAT6Ptyr, acetylase (P300), and deacetylase (SMRTe) binding, histone H3 acetylation, and the presence of RNA Polymerase II on or near the STAT5/STAT6 binding sites in the *IL10* to *PTGS2* enhancer region shown in Figure 3B. Each ChIP multiplex analysis depicted represents the mean of 3 to 5 runs of the analyses on each of control and T1D samples of peripheral blood monocytes. Data is presented on a log scale as an R value = 2^(ΔCt^_nonspecific IgG_^−ΔCt^_specific Ab_^)^ for each specific antibody used [19]. Individual samples (not pooled) were run in at least duplicate and represent data from 5 T1D and 2 Control group members. Due to the low sample size, the apparent trends for high binding of STAT5Ptyr and the other epigenetic modification components seen, when the data were analyzed by ANOVA and pairwise Mann-Whitney U test, no statistically significant differences were found between T1D and control samples for any of proteins examined binding on the *PTGS* enhancer.

Exogenous GM-CSF significantly enhanced SMRT deacetylase associated with its own gene promoter in T1D monocytes compared with the same treatment in control monocytes (p = 0.0040, Mann-Whitney U test, **Figure 4B**). GM-CSF stimulation also significantly increased STAT5Ptyr, STAT6Ptyr, and RNA polymerase II binding at the site in T1D monocytes compared to controls (p = 0.485, p = 0.0283, p = 0.0040, respectively; Mann-Whitney U test). In contrast, GM-CSF treatment of non-autoimmune control monocytes lowered histone acetylation and binding of all the proteins tested at the *CSF2* promoter.

Analysis of STAT5Ptyr binding at the *PTGS2* enhancer region was conducted with the same samples, but low cell/DNA yields from the ChIP isolations only permitted analysis of 5 T1D samples and 2 controls, compromising the statistical analysis of the results. In the samples analyzed, exogenous GM-CSF treatment of T1D monocytes decreased STAT5Ptyr binding but enhanced histone H3 acetylation in T1D monocytes, though neither change was statistically significant. In controls analyzed, GM-CSF decreased SMRT/NCoR and enhanced CBP/P300 binding at the *IL10-PTGS2* region, as well as increased histone H3 acetylation, but these changes were not found to be statistically significant **Figure 5B**).

IL-4 uniformly lowered STAT protein binding at the *CSF2* promoter in both controls and T1D subjects, but did not extinguish it in T1D monocytes (**Figure 4C**). Dexamethasone, a reported inhibitor of STAT protein activated epigenetic modification, inhibited STAT6Ptyr but not STAT5Ptyr binding at the *CSF2* promoter in T1D cells (**Figure 4D**). IL-4 and dexamethasone did not suppress STAT5Ptyr binding, CBP/P300 acetylase binding, histone H3 acetylation, and RNA polymerase II binding at the *IL10 -PTGS2* site in T1D monocytes. Both IL-4 and dexamethasone appeared to promote SMRT binding as seen at the *CSF2* promoter, though these changes were not found to be statistically significant (**Figure 5C–D**).

## Discussion

Mechanistic studies of inflammation activation indicate that changes in epigenetic regulation of genes seen in autoimmune T1D patients may be involved in their immune cell dysfunction [23]–[25]. Genome-wide screens of epigenetic modifications have revealed altered epigenetic control in metabolic and immunologic signaling pathways in T1D compared to non-autoimmune controls [23].

Functional analysis suggests that the resistant STAT5 activation seen in autoimmune human myeloid cells has the potential to alter the epigenetic regulation of at least 2 genes important to promoting inflammation and myeloid APC dysfunction: *CSF2* and *PTGS2* [7], [10]–[15]. In normal monocytes, the binding of STAT5Ptyr and associated epigenetic modification proteins at both *CSF2* and *PTGS2* regulatory sites should be suppressed by the actions of anti-inflammatory, immunosuppressive cytokines such as IL-4 and IL-10. However, IL-4 is known to be inherently low in people at-risk for or with T1D [26]–[29]; and, although IL-10 is not diminished in T1D monocytes, their expression of *PTGS2* remains resistant to its influence [7], [11]. This study finds that epigenetic gene dysregulation in autoimmune cells may be initiated *in vivo* by STAT5 persistent binding at regulatory regions, both for *CSF2* and *PTGS2*. STAT5Ptyr binding at the promoter of *CSF2,* could allow for continued expression of GM-CSF. However, persistence of STAT5Ptyr in T1D monocytes was not dependent on continuous GM-CSF stimulation. Previous studies have shown that GM-CSF can enhance the expression of IL-10- and IL-4- resistant COX2 [6], [7], [10], [12], [20] through STAT5Ptyr-promoted epigenetic dysregulation [6], [8]. Our ChIP analyses suggest that the abundance and persistence of activated STAT5Ptyr found in T1D subject monocytes may be cooperating with STAT6Ptyr at binding sites within the *CSF2* and *PTGS2* regulatory regions. This STAT-STAT interaction may have the potential to promote histone acetylation and transcriptional activation of these genes by interfering with their STAT6Ptyr mediated suppression when little or no activation stimuli are present.

Since STAT5 proteins are adaptor proteins for histone acetylation enzymes, not for methylation enzymes, it is less likely that STAT5Ptyr binding could affect methylation patterns than acetylation modifications. Methylation modifications at these sites were previously reported as unchanged in diabetic monocytes, enhancing the importance of other epigenetic modifications including acetylation for gene dysregulation seen in T1D monocytes [23], [30], [31].

In terms of pharmacological intervention, dexamethasone should have been a promising potential treatment for this defective regulation based on previous reports [13], [14] and its effects seen on the binding of STAT5Ptyr and associated epigenetic modification proteins in control monocytes. However, this drug did not inhibit the STAT5Ptyr binding or epigenetic regulation of gene expression at either the *CSF2* or the *PTGS2* sites in auto-reactive cells. Furthermore, though both dexamethasone and IL-4 alter the amount of CBP/P300 histone acetylase binding and histone H3 acetylation at the *CSF2* promoter in both control and T1D monocytes, neither abolished RNA Polymerase II recruitment at these sites in autoimmune cells, suggesting that gene expression could go forward even in these conditions.

While the data from our study do not prove that GM-CSF induced STAT protein binding causes the epigenetic regulation of *CSF2* and *PTGS2* gene expression, these data support previous findings that STAT5Ptyr activation and binding to epigenetic active sequences are elevated in untreated autoimmune monocytes compared with non-autoimmune monocytes [7], [10], [11], [13], [14]. Our previous work showed no correlation with PGS2 expression with changes in glycemic status [6], [16]; however, it should be noted that blood glucose levels were not concurrently tested with STAT5Ptyr in these studies. Such fluctuations could affect the unstimulated inflammatory responses of the T1D or control individuals examined. Further study of the correlation of inflammation, GM-CSF and STAT5Pytr phenotypes with glycemic state are needed to determine if glycemic control could be involved in STAT5Ptyr dysfunction in T1D.

GM-CSF stimulation of autoimmune monocyte STAT5Ptyr binding is found in direct association with increased binding of enzymes catalyzing histone acetylation/deacetylation, and the presence of RNA Polymerase II at these sites. Altered histone acetylation at these sites in GM-CSF stimulation, suggests that activation of *CSF2* and the inflammation it promotes through *PTGS2,* may be abnormal self-perpetuating in T1D monocytes.
